# Venous thromboembolism risk assessment and prophylaxis in cancer patients

**DOI:** 10.1186/s12885-026-16042-x

**Published:** 2026-04-20

**Authors:** Krish Nebhnani, Salman Muhammad Soomar, Shabeeha Rana

**Affiliations:** 1 King’s College Hospital London, Dubai, UAE; 2 Charles University, Hradec Kralove, Czech Republic

**Keywords:** Venous thromboembolism, Deep Vein Thrombosis, Prophylaxis, Cancer, Oncology

## Abstract

**Background:**

Venous thromboembolism is a potentially life-threatening condition, with patients having active malignancy at a 12-fold higher risk. Hospital-acquired VTE contributes to mortality, morbidity, and prolonged hospitalization. The 2024 British Society for Haematology guidelines recommend VTE risk assessment and prophylaxis within 24 h of admission. This audit evaluated adherence to these guidelines among oncology inpatients, aiming to identify gaps and opportunities for standardizing VTE management.

**Methods:**

A retrospective audit was conducted on 99 oncology inpatients. Electronic records, admission documentation, and drug charts were reviewed for VTE risk assessment completion and timing, prescription and appropriateness of pharmacological prophylaxis, 24-h reassessment, and adherence to cancer-associated thrombosis treatment standards. Patients with documented contraindications to anticoagulation were excluded from adherence analyses. Data were compared with BSH 2024 recommendations, and frequencies and percentages were calculated.

**Results:**

VTE risk assessment was documented in 64.6% (*n* = 64) of patients, while 35.4% (*n* = 35) had no recorded assessment or reassessment. Among assessed patients, 57.8% (*n* = 37) were high risk, 28.1% (*n* = 18) low risk, and 14.1% (*n* = 9) high bleeding risk. Pharmacological prophylaxis was prescribed in 71.7% (*n* = 71) of patients, initiated within 14 h in 36.6% (*n* = 26), and appropriately selected in 88.5% (*n* = 23) of those receiving timely prophylaxis. Reassessment within 24 h occurred in 12.1% (*n* = 12) of patients. Hospital-acquired thrombosis was identified in 6.1% (*n* = 6) of patients, with prophylaxis adherence varying across services: 71.0% (*n* = 71) in medical oncology, 26.2% (*n* = 26) in surgical oncology, and 11.1% (*n* = 11) in myeloma pathways. Catheter-related thrombosis prevention was documented in 4.0% (*n* = 4). For CAT management, 69.6% (*n* = 69) received anticoagulation during the first six months and continued beyond six months if the malignancy remained active. Recurrent VTE occurred in 22.2% (*n* = 22) of patients receiving complete treatment.

**Conclusion:**

Clinical practice in this oncology department largely aligns with BSH 2024 recommendations; however, documentation is inconsistent and incomplete. Standardized electronic templates, mandatory assessment fields, clarified myeloma pathways, and targeted staff education may improve adherence. A follow-up re-audit is recommended to evaluate progress and reinforce guideline implementation.

## Introduction

Venous thromboembolism (VTE) refers to the occurrence of thrombus in the venous system, inclusive of Deep Vein Thrombosis (DVT) and Pulmonary Embolism (PE) [1]. DVT usually occurs in the veins of the lower limbs, pelvis, or thigh, although upper limbs may also be involved [2]. A DVT may embolize, giving rise to pulmonary embolism, a condition that affects the pulmonary vasculature, exerting strain on the right ventricle due to its load, thus producing effects on both the oxygenation and functions of the right side of the heart. DVT may progress, leading to the subsequent development of the post-thrombotic syndrome, taking the form of pain, inflammation, as well as being a contributor to high morbidity [3].

Cancer-associated thrombosis (CAT) is thus a severe contributor to morbidity and mortality rates in oncology patients, being the second most frequent cause of death in this group, the first being progression of the disease. Compared with the risk of VTE within the general population, cancer patients experience a risk that is 4- to sevenfold higher, although some forms of malignancies, such as pancreatic, gastric, lung, ovarian, or hematological cancers, increase the risk [4].

CAT arises from complex interactions between tumor, host, and therapeutic variables. Tumor cells promote hypercoagulability through the activation of the coagulation pathway via tissue factor, as well as cancer cell procoagulant activity, initiating thrombin and fibrin production [5]. Inflammatory mediators (IL-1β, IL-6, and TNF-α) increase endothelial cell activation and further enhance hypercoagulability through platelet activation [6]. Moreover, tumor cell-secreted extracellular vesicles also play a role in thrombus formation through phospholipid promotion of coagulation [7]. Finally, cell necrosis caused by hypoxia promotes hypercoagulability through the production of genes involved in thrombus formation, as well as through the inflammatory pathways involved in the production of neutrophils [6, 8]. Factors like endothelial damage, venous stasis, immobility, central venous catheters, or large tumor masses meet Virchow’s triad, thus increasing the risk of thrombosis [9]. Antineoplastic medication, such as chemo pharmaceutical drugs, hormonal drugs, and antiangiogenic drugs, further increases this risk through endothelial damage [10].

Oncologic patients face a significantly increased risk of VTE because of hypercoagulability associated with cancer, as well as the consequences of hospitalization, surgery, immobilization, and systemic treatments. Incidents of VTE were found, through a meta-analysis, of approximately 13 per 1,000 person-years in cancer patients, with a risk that was found to be fourfold higher than that of the general population, with the highest rates being found within patients with pancreatic, brain, gastric, lung, and hematological malignancies [11]. VTE is one of the most frequent causes of death in patients with malignancies, for whom frequent risk assessment for VTE, as well as thromboprophylaxis, is recommended [12]. Evidence from real-world clinical settings consistently demonstrates poor adherence with recommended practices for venous thromboembolism (VTE) prevention, particularly among hospitalized patients and those with cancer. Previous audits indicate that fewer than 60% of cancer patients have a documented VTE risk assessment, contributing to preventable patient mortality and increased healthcare costs. This gap in adherence may be driven by factors such as the lack of standardized documentation systems, variability in clinician awareness, the complexity of risk assessment tools, and competing priorities in acute care settings [2]. Pharmacological prophylaxis, specifically LMWH or DOAC, is effective, safe, yet still not uniformly practiced. variability based on cancer type and bleeding risk [13].

Although thromboprophylaxis for VTE in cancer patients is strongly recommended in established clinical guidelines, its implementation remains inconsistent and is often not extended beyond hospital discharge globally. Findings from the ENDORSE study, conducted across 32 countries, further highlight this issue, showing that only about half of hospitalized patients at risk of VTE received appropriate thromboprophylaxis, with notable geographic variation [3]. Finally, within the DIONYS Registry of patients with abdominal or pelvic surgery, as well as cancer surgery, in Latin America, Africa, and the Middle East, the rate of full ACCP guideline adherence (regarding drug, dose, as well as duration) was a mere 12.3%, entirely because of inadequate use of extended thromboprophylaxis beyond hospital discharge [14].

The British Society for Haematology (BSH) 2024 guidelines recommend that all hospitalized cancer patients undergo formal VTE risk assessment within 24 h of admission, with stratification into VTE and bleeding risk categories. Pharmacological thromboprophylaxis is advised for patients at high VTE risk without contraindications, with reassessment within 24 h and continuation based on clinical status. For confirmed CAT, anticoagulation is recommended for at least 6 months and extended if malignancy remains active. While previous studies have demonstrated suboptimal adherence, this audit provides institution-specific insights, identifies documentation gaps, and highlights actionable system-level improvements.

This audit aimed to evaluate adherence with VTE risk assessment and prophylaxis guidelines in Oncology patients at King's College Hospital London, Dubai, in line with the 2024 British Society for Haematology (BSH) guidelines. The aim was to identify deficiencies in risk assessment, documentation, and prophylaxis, and inform improvements in the oncology service. Based on institutional audit standards, ≥ 80% adherence with VTE risk assessment documentation was considered indicative of good clinical practice.

## Methods

### Study design

This was a retrospective clinical audit conducted in King's College Hospital London, Dubai. The application of the 2024 British Society for Hematology guidelines on venous thromboembolism risk assessment and prophylaxis in real-world settings among oncology patients was assessed. Data were extracted from electronically stored medical records of eligible admissions.

### Study duration

Patient admissions occurred between January 2023 and October 2025, while data extraction was conducted from October to December 2025.

### Study population

All adult patients aged ≥ 18 years with a clear diagnosis of active malignancy who were admitted during the study time were included. Patients with pre-existing VTE or already receiving therapeutic anticoagulation were identified and analyzed separately where relevant.

Patients admitted for palliative care purposes were excluded, defined as those receiving end-of-life care where thromboprophylaxis was not clinically indicated. Also, records that were incompletely documented and no diagnosis of malignancy were not included.

### Sampling and sample size

A simple random sampling was performed. As this study was a retrospective clinical audit, formal sample size calculation was not performed. A sample of 99 records was selected pragmatically to provide a representative snapshot of clinical practice while ensuring feasibility of detailed manual data extraction. Patient records were selected using a structured sampling frame derived from eligible admissions during the study period.

### Data collection and variables

Data was collected by trained clinical reviewers and cross-checked by the second investigator to ensure accuracy and consistency. Data were collected on pre-designed Performa. Data about VTE risk assessment, reassessment, prophylaxis type and adherence to the BSH 2024 guideline were collected from a review of patient files. Symptoms of patients were considered for VTE diagnosis to make sure the BSH 2024 guidelines were followed. Covariates were the location of cancer, treatment regimen, risk of bleeding, and comorbid disease. The main outcome was the percentage of oncology patients with VTE risk assessment recorded and appropriate prophylactic management. Appropriate pharmacological prophylaxis was assessed based on the drug choice, duration, and frequency prescribed to the patients. No interventions preceded or followed the data collection period.

### Data analysis

Mean and Standard Deviation (SD) were calculated for the quantitative normally distributed variables. The Shapiro–Wilk test was used for determining normality. Frequency and percentages were calculated for the categorical variables. Adherence was presented in percentages, and bar graphs were developed to visually display the data.

## Results

### VTE risk assessment and prophylaxis

**Fig. 1:**
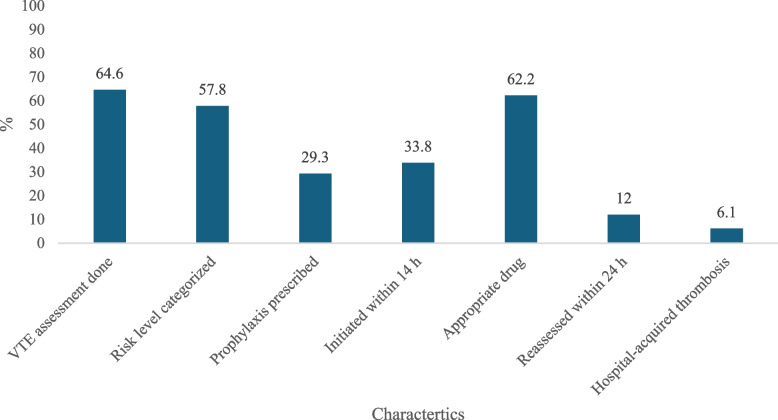
Adherence with VTE risk assessment and prophylaxis standards (*n* = 99)

A total of 99 oncology patients were included in the audit, with a mean age of 49.4 years (SD ± 11.8). Females comprised 59.6% of the study population (*n* = 59), while males accounted for 40.4% (*n* = 40). VTE risk assessment was documented on admission in 64.6% of patients (*n* = 64), whereas 35.4% (n = 35) had no formal assessment recorded. Among those assessed, 57.8% (*n* = 37) were classified as high risk for VTE, 28.1% (*n* = 18) as low risk, and 14.1% (*n* = 9) as high risk with concurrent high bleeding risk. Pharmacological prophylaxis was prescribed in 71.7% of patients (*n* = 71). Among these, initiation within 14 h occurred in 36.6% (*n* = 26), while 62.0% (*n* = 44) did not receive timely initiation, and timing was not specified for 1.4% (*n* = 1). Of the patients who received prophylaxis within 14 h (*n* = 26), 88.5% (*n* = 23) received the appropriate anticoagulant, while 11.5% (*n* = 3) received an inappropriate drug. Reassessment of VTE risk within 24 h was documented in 12.1% of patients (*n* = 12), not performed in 21.2% (*n* = 21), and not specified in 66.7% (*n* = 66). Hospital-acquired thrombosis (HAT) occurred in 6.1% of patients (*n* = 6). Among those who developed HAT, 100% (*n* = 6) had received appropriate thromboprophylaxis (Table 1, Fig. 1).

**Table 1:** Baseline characteristics, VTE risk assessment, and prophylaxis data among cancer patients (*n* = 99)

Characteristics	*n* (%)
Age (Mean ± SD), years	49.4 ± 11.8
Gender	
Male	40 (40.4)
Female	59 (59.6)
VTE Risk Assessment on Admission	
Yes	64 (64.6)
No	35 (35.4)
VTE Risk Level (*n* = 64)	
High risk	37 (57.8)
Low risk	18 (28.1)
High risk with high bleeding risk	9 (14.1)
Pharmacological Prophylaxis Prescribed	
Yes	71 (71.7)
No	28 (28.3)
Among those prescribed prophylaxis (*n* = 71)	
Initiated within 14 h	26 (36.6)
Not initiated within 14 h	44 (62.0)
Not specified	1 (1.4)
Among those initiated within 14 h (*n* = 26)	
Appropriate drug prescribed	23 (88.5)
Inappropriate drug prescribed	3 (11.5)
Reassessment within 24 h (*n* = 99)	
Yes	12 (12.1)
No	21 (21.2)
Not specified	66 (66.7)
Hospital-Acquired Thrombosis (HAT)	
Yes	6 (6.1)
No	93 (93.9)
Among patients with HAT (*n* = 6)	
Received appropriate thromboprophylaxis	6 (100.0)
Did not receive appropriate thromboprophylaxis	0 (0.0)

### Adherence with BSH 2024 CAT standards

**Fig. 2:**
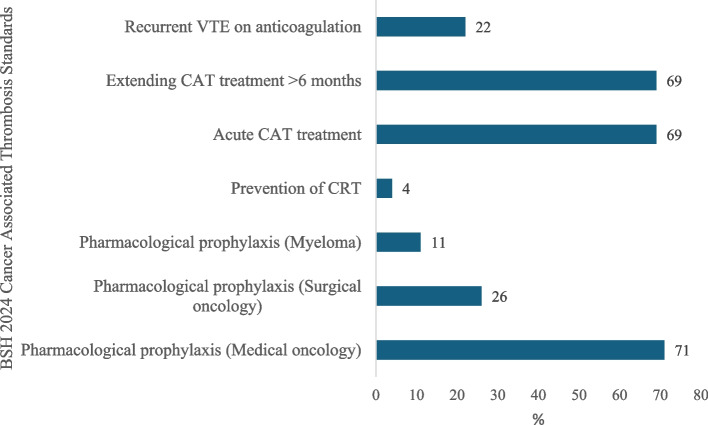
Adherence with BSH 2024 VTE risk assessment guidelines (*n* = 99)

Pharmacological prophylaxis was administered to 71.7% of medical oncology patients (*n* = 71), whereas only 26.2% of surgical oncology patients (*n* = 26) received prophylaxis. Myeloma-specific risk assessment was documented in 11.1% of patients (*n* = 11). Preventive measures for catheter-related thrombosis (CRT) were implemented in a small proportion of cases (4.0%, *n* = 4). Treatment for cancer associated thrombosis during the first six months was provided to 69.6% of patients (*n* = 69), and a similar proportion (69.6%, *n* = 69) continued anticoagulation beyond six months. Recurrent venous thromboembolism while on anticoagulation therapy was observed in 22.2% of patients (*n* = 22) (Table 2, Fig. 2).

**Table 2:** Compliance with BSH 2024 guidelines for cancer-associated thrombosis (*n* = 99)

*Characteristics*	*n (%)*
Pharmacological prophylaxis (Medical oncology patients)	71 (71.7)
Pharmacological prophylaxis (Surgical oncology patients)	26 (26.2)
Pharmacological prophylaxis (Myeloma-specific risk assessed)	11 (11.1)
Prevention of catheter-related thrombosis (CRT)	4 (4.0)
Acute CAT treatment (first 6 months)	69 (69.6)
Extending CAT treatment (> 6 months)	69 (69.6)
Recurrent VTE on anticoagulation	22 (22.2)

## Discussion

This audit evaluated adherence to the 2024 British Society for Haematology guidelines for the prevention and treatment of venous thromboembolism in cancer patients within an oncology unit. Overall, while clinical practice aligns partially with guideline recommendations, several gaps were identified, particularly in the standardization and documentation of VTE risk assessment and thromboprophylaxis.

A documented VTE risk assessment was available in 64.6% of admissions. While this rate may appear moderate, it is difficult to define it as “good” compliance given the absence of a standardized benchmark. For context, global audits have consistently shown that VTE risk assessment in cancer patients is variably documented, ranging from 50 to 75% [15]. For instance, in a UK-wide audit of medical admissions, approximately 65% of patients had documented VTE risk assessments despite national guidelines (National VTE Prevention Programme). Similar trends have been observed across Europe and North America, where prophylactics are generally prescribed but formal risk assessment is often inconsistently completed [16].

Among patients with documented assessments, 57.8% were classified as high risk, supporting evidence that cancer patients are a particularly thrombogenic population. Previous studies have reported 40–60% of hospitalized oncology patients as high risk, underscoring the critical need for risk stratification at admission. The absence of documentation therefore reflects missed opportunities for standardized care rather than lack of physician awareness [17].

Significant discrepancies in documentation were noted across specialties. Medical oncology patients had higher rates of documented risk assessment (71%) compared with surgical oncology and myeloma patients [18]. International literature mirrors these findings, with medical departments often showing better adherence than surgical services, likely due to differences in operative workflows, competing clinical priorities, and the use of individualized risk assessment charts [18]. In myeloma, tools such as IMPEDE-VTE and SaVED are infrequently utilized, likely due to their complexity [19].

As reported in previous studies, low recorded adherence does not always indicate suboptimal care. Patients with gastrointestinal malignancies, thrombocytopenia, or active bleeding are appropriately excluded from pharmacological prophylaxis, as per guideline recommendations. In many audits, such clinical decisions are not adequately documented, resulting in apparent non-adherence [16]. In this study, omissions in recording may similarly have contributed to the observed gaps.

Management of cancer-associated thrombosis was generally consistent with guideline recommendations. Approximately 70% of patients received optimal anticoagulation during the initial six months and continued therapy beyond six months while the malignancy remained active. This aligns with real-world studies showing 65–75% adherence to anticoagulation in oncology populations following the introduction of DOAC guidelines [17]. Nevertheless, recurrent VTE was observed in 22% of treated patients, comparable with published rates of 15–25% [9].

A key limitation highlighted in this study, consistent with international literature, is fragmented documentation. Non-mandatory or scattered electronic fields contribute to gaps in recorded adherence. Institutions with mandatory electronic VTE risk assessment fields have demonstrated improved documentation rates, although prescribing practices may remain unaffected [20].

Compared to international standards, adherence to VTE prophylaxis and CAT management in this audit compares favorably. However, challenges in documentation mirror those seen globally. Improving adherence likely depends more on standardized recording of risk assessments than on prescribing itself. Interventions such as enhanced electronic recording systems, mandatory risk assessment fields, and targeted staff education have been shown to improve documentation. A follow-up re-audit would provide an objective measure of improvement and reinforce alignment with the BSH 2024 guidelines. Implementation of standardized protocol-based VTE assessment tools may improve adherence, and future pre–post studies evaluating such interventions are warranted. Recurrent VTE was not assessed during the available follow-up period.

### Strengths and limitations

This study provides valuable insights into the implementation of VTE risk assessment and thromboprophylaxis in an oncology setting, highlighting areas for improvement in clinical practice. The inclusion of 99 patients allowed for the evaluation of VTE risk and management across a diverse population, providing a practical understanding of adherence patterns and potential gaps within a hospital department. Additionally, the study assessed real-world compliance with the 2024 British Society for Haematology guidelines, offering a foundation for future quality improvement initiatives.

Several limitations should be considered. The study’s small sample size (*n* = 99) and retrospective design may limit the generalizability of the findings and the completeness of documentation. It was conducted at a single center, further restricting generalizability to other institutions or healthcare settings. There was no post-discharge follow-up, which could lead to underestimation of the true incidence of VTE. The dataset did not include bleeding complications, preventing assessment of the safety of pharmacological prophylaxis or full adherence to guideline recommendations. Furthermore, the study did not utilize a validated VTE risk assessment model (RAM), such as the PADUA score, limiting the evaluation of whether prophylaxis was appropriately tailored to individual patient risk. Finally, a proportion of patients were admitted prior to the publication of the BSH 2024 guidelines, so lower adherence rates during this period may reflect historical practices rather than current guideline compliance.

## Conclusion

This audit demonstrates that the oncology department generally adheres to the 2024 BSH guidelines for VTE prevention and treatment, particularly when pharmacological prophylaxis and cancer-associated thrombosis management are properly documented. However, significant limitations in documentation prevent a fully quantifiable assessment of guideline adherence. Recurrent VTE observed in a subset of patients highlights the inherent complexities of managing thrombosis in the context of active cancer. The primary opportunities for improvement lie in system-level interventions rather than changes to clinical practice. Implementing standardized electronic templates, mandating completion of risk assessment fields, and conducting targeted staff education can enhance documentation quality, ensure transparency, and facilitate reliable assessment of adherence to guidelines. While the study’s small sample size, single-center design, retrospective approach, and lack of a validated risk assessment model limit generalizability, the findings underscore the importance of structured documentation in improving patient care and provide actionable insights for future quality improvement initiatives.

## Data Availability

The datasets used and/or analyzed during the current study are available from the corresponding author on reasonable request.

## Abbreviations

VTE: Venous Thromboembolism
DVT: Deep Vein Thrombosis
CAT: Cancer-Associated Thrombosis
LMWH: Low Molecular Weight Heparin
DOAC: Direct Oral Anticoagulants
BSH: British Society for Haematology
ASCO: American Society of Clinical Oncology
ESMO: European Society for Medical Oncology
NET: Neutrophil Extracellular Trap
CRT: Catheter-Related Thrombosis
